# Pair combinations of human monoclonal antibodies fully protected mice against bunyavirus SFTSV lethal challenge

**DOI:** 10.1371/journal.ppat.1012889

**Published:** 2025-01-31

**Authors:** Bang Li, Xiang-rong Qin, Jia-chen Qu, Guan-du Wu, Wen-kang Zhang, Ze-zheng Jiang, Pan-pan Liu, Ze-min Li, Tian-mei Yu, Chuan-min Zhou, Yong-jun Jiao, Xue-jie Yu

**Affiliations:** 1 State Key Laboratory of Virology, School of Public Health, Wuhan University, Wuhan, China; 2 Department of Clinical Laboratory, The Second Hospital of Shandong University, Jinan, China; 3 Department of Critical Care Medicine, Zhongnan Hospital of Wuhan University, Wuhan, China; 4 Institute of Pathogenic Microbiology, Jiangsu Provincial Center for Disease Prevention and Control, Nanjing, China; University of New Mexico School of Medicine, UNITED STATES OF AMERICA

## Abstract

Severe fever with thrombocytopenia syndrome (SFTS) is a viral hemorrhagic fever caused by a tick-borne virus SFTSV with a mortality rate of up to 30%. Currently, there is no vaccine or effective therapy for SFTS. Neutralizing monoclonal antibody therapy, which provides immediate passive immunity and may limit disease progression, has emerged as a reliable approach for developing therapeutic drugs for SFTS. In this study, 4 human monoclonal antibodies (hmAbs) derived from convalescent SFTS patients’ lymphocytes based on human single-chain variable fragment antibody libraries were tested for their neutralizing activities in cells and their treatment effect in animals individually and in pair combinations. The neutralization test showed that all 4 hmAbs exhibited strong neutralizing activity against SFTSV infection *in vitro*. The protection rate of hmAbs 4-6, 1F6, 1B2, and 4-5 against SFTSV lethal challenge in *IFNAR1*^-/-^ A129 mice are 50%, 16.7%, 83.3%, and 66.7%, respectively. Notably, the pair combination of antibodies (1B2 and 4-5, 1B2 and 1F6) that recognized distinct epitopes protected 100% of mice against SFTSV lethal challenge. In conclusion, our findings indicate that the pair combinations of hmAbs 1B2 and 4-5 or hmAbs 1B2 and 1F6 may serve as promising therapeutic drugs for treating SFTSV infection.

## Author summary

Severe fever with thrombocytopenia syndrome (SFTS) is a viral hemorrhagic fever in East and Southeast Asia countries with a mortality rate from 16% to 30%. The disease is caused by a bunyavirus, SFTSV and transmitted through tick bite. There is no vaccine or effective therapy for the disease. Monoclonal antibodies are increasingly becoming a reliable method of treating viral infections. In this study, we tested the neutralization activity on cell culture and protectivity against a lethal challenge in mice of 4 human monoclonal antibodies (hmAbs 4-6, 1F6, 1B2, and 4-5). The hmAbs recognized Gn, a viral coat protein of SFTSV. Our results showed when the hmAbs are individually used, they all can neutralize SFTSV infection in cell culture and conferred partial protection for mice against lethal dose challenge of the virus. Whereas when two hmAbs recognizing distinct epitopes were combined together to use, the pair-combinations of hmAbs 1B2 and 4-5 or hmAbs 1B2 and 1F6 provided complete protection against SFTSV lethal challenge in mice. We anticipate that these antibody cocktails may be developed into a new therapy for treating SFTS.

## Introduction

Severe fever with thrombocytopenia syndrome virus (SFTSV) is a negative-sense RNA virus in the genus *Bandavirus* in the family *Phenuiviridae*, order *Bunyavirales* [1]. SFTSV was first reported in 2011 in China and subsequently reported in other Asian countries, including South Korea, Japan, Thailand, and Vietnam [2-6]. SFTSV is highly infectious for human beings causing a viral hemorrhagic fever, termed severe fever with thrombocytopenia syndrome (SFTS), with a high mortality rate from 16% to 30% in East Asia countries [2,7]. The clinical features of SFTS were mainly characterized by fever, thrombocytopenia, leukocytopenia, vomiting, diarrhea, and hemorrhage, and patients may die of multiple organ failure [2-5]. In 2018, SFTSV was listed as a priority pathogen by the World Health Organization [8]. SFTSV is a tick-borne virus, which has been isolated or detected from *Haemaphysalis longicornis*, *H. flava*, *H. formosensis*, *H. hystricis*, and *H. megaspinosa*, and *Amblyomma testudinarium* tick species [2,9]. *Haemaphysalis longicornis* and *H. flava* have been laboratory-confirmed to effectively transmit SFTSV [10,11]. It is occasionally transmitted from persons or animals to humans via contact with the body fluids of SFTS patients and probable aerosol [12-15].

The SFTSV genome consists of three negative RNA segments. The M segment encodes the envelope glycoprotein (GP) cleaved into Gn and Gc, which are crucial for SFTSV invasion, facilitating receptor attachment, viral entry, virion assembly, and exocytosis by utilizing autophagic vesicles [16]. The increasing number of human cases, the rapid global dissemination of tick vectors, the emergence of new genotypes, and the recurrent instances of human-to-human transmission have raised concerns regarding a potential SFTS pandemic [17-19]. However, there was no licensed vaccine or neutralizing antibody available at the moment for combating SFTS attacks.

Monoclonal antibodies (mAbs) generally inhibit viral infection by precisely recognizing and binding specific epitopes, enhancing phagocytosis by opsonization, and destroying infected cells [20]. Various studies elicited that SFTSV Gn and Gc might be specific targets for developing vaccines and therapeutic mAbs [21-23]. The convalescent sera of SFTS patients were found effective for SFTS treatment [24]. Hence, considering the large number of SFTS patients, mAbs, which were more economical and quicker to develop, were essential for treating SFTS. Several neutralizing mAbs (Ab10, SNB02, and 40C10) that recognized different epitopes targeted in SFTSV Gn have been reported, which displayed neutralization activity both *in vitro* and *in vivo* [25-27]. Recent advancements in bispecific antibodies engineering using two human monoclonal antibodies (hmAbs) SF5 and SF83, which recognize Gn and Gc and exhibit an enhanced protection efficacy *in vivo* compared to the parental mAbs [28], underscore the need for further exploration of highly protective hmAbs or a combination of hmAbs targeting distinct epitopes to treat SFTSV infection. Previous studies have shown that antibody cocktail therapy showed a cooperative effect against various viral infections compared to single mAbs in clinical trials [29-31]. Hence, developing antibody cocktails for blocking SFTSV infection and SFTS treatment is equally important.

This study aimed to test the neutralization and productive effects of 4 anti-SFTSV Gn hmAbs 4-6, 1F6, 1B2, and 4-5 in cells and animals to find therapeutic monoclonal antibodies and antibody cocktails that can be used to treat SFTSV infection.

## Results

### Production and identification of hmAbs to SFTSV Gn

**Fig 1:**
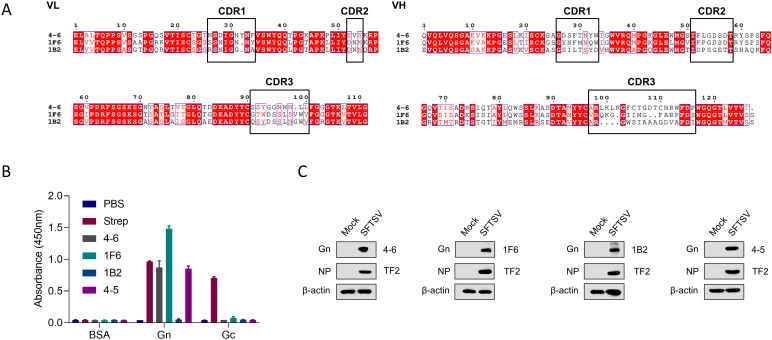
Production and identification of hmAbs to SFTSV Gn. (A) The amino acid sequences of VL and VH domains of hmAbs. The nucleotides in the red boxes are conserved among the 3 hmAbs and dots represent gaps. Black boxes indicate complementary determining regions (CDR) of each variable region defined by the International Immunogenetics Information System (IMGT). (B) ELISA analysis of the binding ability of hmAbs (0.1 µg/mL) to recombinant protein Gn, Gc, or bovine serum albumin (BSA) pre-coated ELISA plates. Strep was used as a positive control. (C) Vero cells were infected with 1 MOI of SFTSV and the protein levels of SFTSV Gn and NP were analyzed with Western blot. TF2, an anti-SFTSV NP-specific mAb.

We constructed a phage-displayed human single-chain variable fragments (scFv) antibody library using human lymphocytes isolated from 5 convalescent SFTS patients. The phage-displayed library was subjected to four rounds of biopanning against the purified recombinant Gn protein (S1A Fig). The individual phage colonies were tested for binding to purified recombinant Gn protein in a phage enzyme-linked immunosorbent assay (ELISA). Positive clones were selected and the genes of VH and VL chains were sequenced to determine the scFv nucleotide sequence. We successfully obtained 3 unique clones reactive to Gn, namely 4-6, 1F6, and 1B2. The 3 scFv were encoded by 3 different VH and VL sequences (Fig 1A). Then, the paired VH and VL were cloned into the pCAGGS expression vector as full-length IgG4 antibodies, expressed in HEK293T cells, and purified (S2A-S2C Fig). The 3 hmAbs 4-6, 1F6, 1B2 produced in this study and a hmAb 4-5 reported by one of us previously [32] were tested for the binding to SFTSV glycoprotein Gn and Gc, respectively. ELISA assays indicated that all 4 hmAbs did not react with Gc (Fig 1B). ELISA showed that hmAbs 4-6, 1F6, and 4-5 can bind to Gn, while 1B2 did not (Fig 1B). However, all the hmAb 4-6, 1F6, 1B2, and 4-5 demonstrated the ability to recognize the SFTSV Gn protein with Western blot (Fig 1C).

### hmAbs derived from Gn neutralizing SFTSV infection *in vitro*

**Fig 2:**
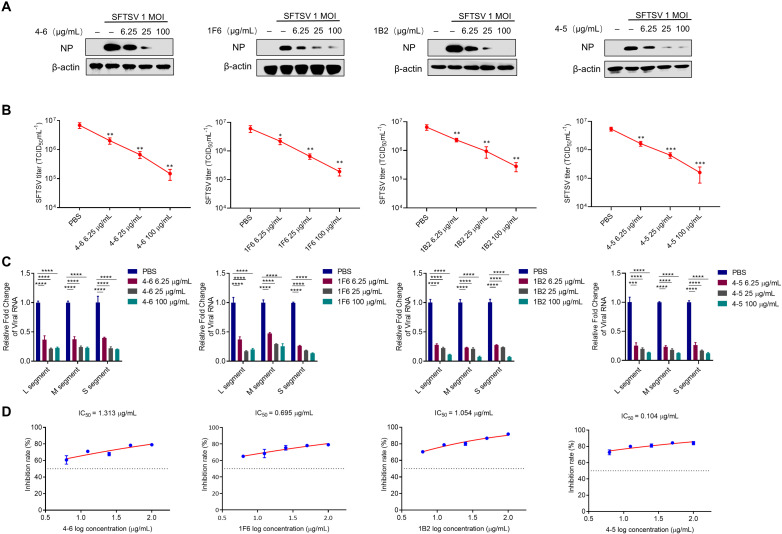
hmAbs derived from Gn neutralizing SFTSV infection *in vitro.* (A-C) Different doses of each hmAb were premixed with SFTSV (1 MOI) at 37°C for 1 h, and the mixture was incubated with cells for 2 h, and then the supernatant was replaced with 2% maintenance medium and cultured at 37°C for 24 h. Western blot analysis was used to determine the protein level of SFTSV NP. PBS was used as negative control. (B) SFTSV titer in the supernatant of infected cells was measured with TCID_50_ assays at 24 h post-infection. (C) Different doses of each hmAb were premixed with 100 TCID_50_ SFTSV at 37°C for 1 h, and the mixture was incubated with cells for 2 h, and then the supernatant was replaced with 2% maintenance medium and cultured at 37°C for 24 h. RT-qPCR was used to determine the viral RNA level of SFTSV L/M/S segments. (D) Different doses of each hmAb (6.25, 12.5, 25, 50, and 100 µg/mL) were premixed with 100 TCID_50_ SFTSV at 37°C for 1 h, and the mixture was incubated with cells for 2 h, and then the supernatant was replaced with 2% maintenance medium and cultured at 37°C for 24 h. RT-qPCR was used to detect the viral RNA level of SFTSV. IC_50_: 50% inhibitory concentration. Data were obtained from three independent experiments (n = 3) and were analyzed with a two-tailed Student’s *t*-test. Data are presented as mean ± standard deviation (SD). *, *P* < 0.05; **, *P* < 0.01; ***, *P* < 0.001; ****, *P* < 0.0001; ns, no significance.

SFTSV proliferates efficiently in Vero cells, which are commonly used to test the antiviral effect of drugs. To validate the neutralizing activity of the hmAbs against SFTSV (strain JS2011-013-1), each hmAb was incubated with SFTSV for 1 h at 37°C before adding to Vero cells. Subsequently, a comprehensive assessment was conducted with Western blot, and real-time quantitative PCR (RT-qPCR) to evaluate the effectiveness of neutralization. As shown in Fig 2A and 2C, decreased SFTSV NP expression and RNA level of L/M/S segments were observed in each hmAb treated cells in a dose-dependent manner. Consistently, we observed that the virus titer decreased from 10^6.67^–10^6.9^ TCID_50_/mL to 10^4.8^–10^5.59^ TCID_50_/mL with 100 µg/mL of hmAbs treatment (Fig 2B). Half-maximal inhibitory concentration (IC_50_) values of hmAbs 4-6, 1F6, 1B2, and 4-5 were 1.313 µg/mL, 0.695 µg/mL, 1.054 µg/mL, and 0.104 µg/mL, respectively (Fig 2D). Three SFTSV strains from clades II, III, and IV (HNXY2017-66, HBMC16_human_2015, and HNXY2017-50) [33] were used to evaluate the broad neutralization ability of the hmAbs. The results demonstrated that all the hmAbs effectively neutralized the 3 genotypes of SFTSV strains (S3A–S3C Fig). Taken together, these data indicate that hmAbs 4-6, 1F6, 1B2, and 4-5 all exhibit strong neutralizing activity to various genotypes of SFTSV by recognizing its Gn specifically.

### Potency of a single human monoclonal antibody to neutralize SFTSV infection

**Fig 3:**
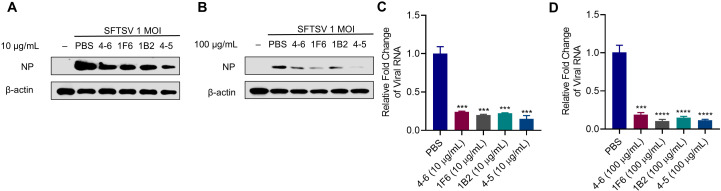
Potency of a single human monoclonal antibody to neutralize SFTSV infection. (A-D) Effects of different antibodies on SFTSV infection at the concentration of 10 µg/mL or 100 µg/mL, respectively. (A-B) The hmAbs were premixed with SFTSV (1 MOI) at 37°C for 1 h, the mixture was incubated with cells for 2 h, and then the supernatant was replaced with 2% maintenance medium and cultured at 37°C for 24 h. Western blot was used to determine the protein level of SFTSV NP. (C-D) The hmAbs were premixed with 100 TCID_50_ SFTSV at 37°C for 1 h, the mixture was incubated with cells for 2 h, and then the supernatant was replaced with 2% maintenance medium and cultured at 37°C for 24 h. RT-qPCR was used to analyze the viral RNA level of SFTSV. PBS was used as negative control. Data were obtained from three independent experiments (n = 3) and were analyzed with a two-tailed Student’s *t*-test. Data are presented as mean ± standard deviation (SD). *, *P* < 0.05; **, *P* < 0.01; ***, *P* < 0.001; ****, *P* < 0.0001; ns, no significance.

We evaluated the neutralizing activity of the 4 hmAbs (4-6, 1F6, 1B2, and 4-5) against SFTSV. At 10 µg/mL, hmAbs 4-6, 1B2, and 1F6 reduced viral RNA levels by 76%, 77.7%, and 80% respectively, hmAb 4-5 demonstrated markedly stronger neutralizing activity against SFTSV, achieving a remarkable reduction in viral RNA levels by 85% (Fig 3A and 3C). At a higher concentration (100 µg/mL), all hmAbs showed increased neutralizing activity with reduction of viral RNA levels by 81% (hmAb 4-6), 85% (1B2), 88.3% (4-5), and 89.3% (1F6), respectively (Fig 3B and 3D). Taken together, these findings suggest variations in the neutralizing activity of the 4 hmAbs across different concentrations, with 1F6 and 4-5 exhibiting stronger neutralizing activity *in vitro* compared to 4-6 and 1B2.

### hmAbs neutralize SFTSV binding and internalization

**Fig 4:**
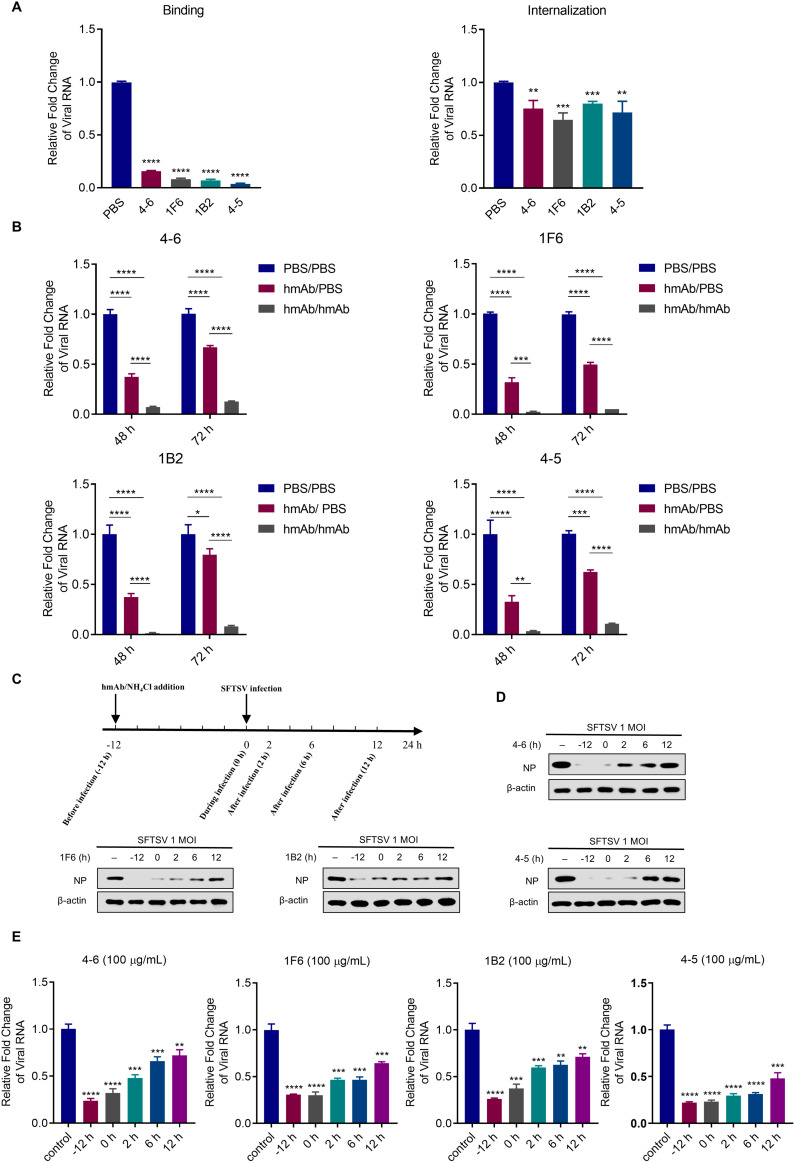
hmAbs neutralize SFTSV binding and internalization. (A) Effect of hmAbs on the binding and internalization of SFTSV. For the binding assay: viruses (1 MOI) were first premixed with hmAbs (100 µg/mL) or PBS at 37°C for 1 h. This mixture was then added to Vero cells at 4°C. After 1 h, the mixture was removed and the cells were washed, and the relative viral RNA level of SFTSV was measured to assess the binding efficiency. For internalization assay, SFTSV (1 MOI) was incubated with cells at 4 °C for 1 h. Then, 100 µg/mL of hmAbs were added to cells, and cells were incubated at 37 °C for 2 h to allow viral entry into cells. Cell surface-bound virions were then removed by trypsin treatment, and relative viral RNA level of internalized SFTSV was measured. (B) Each hmAb (25 µg/mL) or PBS control was premixed with 100 TCID_50_ SFTSV at 37°C for 1 h, and the mixture was incubated with cells for 2 h, and then the supernatant was replaced with replacement medium with hmAb (25 µg/mL) (hmAb/hmAb) or without hmAb (hmAb/PBS) and was cultured at 37°C for 48–72 h. RT-qPCR was used to determine the viral RNA level of SFTSV. (C) Schematic timeline of hmAb treatment on SFTSV. hmAbs or NH4Cl was added to cells 12 h before and 0, 2, 6, or 12 h after SFTSV (1 MOI) infection. (D) 100 µg/mL of hmAbs were added to cells before SFTSV infection (−12 h), at the same time as SFTSV infection (0 h), or after SFTSV infection (2, 6, and 12 h) at different time points. The cells were harvested 24 h after SFTSV infection and the protein level of SFTSV NP were analyzed with Western blot. (E) Total cell RNA was extracted 24 h after SFTSV infection. RT-qPCR was used to determine the viral RNA level of SFTSV. Data were obtained from three independent experiments (n = 3) and were analyzed with a two-tailed Student’s *t*-test. Data are presented as mean ± standard deviation (SD). *, *P* < 0.05; **, *P* < 0.01; ***, *P* < 0.001; ****, *P* < 0.0001; ns, no significance.

To determining the mechanism by which hmAbs neutralize SFTSV infection, we conducted virus binding and internalization experiments. The results showed that hmAbs significantly inhibited both viral binding and internalization, with reductions ranging from 84.3% to 96.3% and from 20% to 35.3%, respectively (Fig 4A). We then determined whether the hmAbs prevent the spread of the progeny virus in cell culture. Culture medium was replaced with replacement medium containing the hmAb or without the hmAb at 48 h and 72 h after hmAb treatment. We found that replacement medium containing hmAb was more effective in inhibiting virus replication than the replacement medium without hmAb. Replacement medium containing the hmAb reduced viral RNA levels by 92% to 99% and 87% to 95% at 48 h and 72 h, respectively, whereas the replacement medium without hmAb reduced viral RNA levels by 62.7% to 68% and 20.3% to 50.3% at the same time points (Fig 4B). Next, time-dependent treatment with 100 µg/mL of hmAb was used to study the optimal time point for hmAb inhibition of SFTSV infection. SFTSV-infected Vero cells were treated with hmAb at 12 h before infection, 0, 2, 6, or 12 h after infection, and NH_4_Cl was used as positive control (Fig 4C). Western blot and RT-qPCR showed that all hmAbs were able to inhibit SFTSV-infecting cells at all-time points from 12 h before infection to 12 h after infection. The SFTSV replication is negatively correlated with hmAb treatment time, that is, the earlier the treatment, the less the amount of SFTSV NP and viral RNA (Figs 4D, 4E, and S4). The best treatment time points in neutralizing SFTSV infection were 12 h before infection and at the time of infection. The hmAb 4-5 had the best effect on the inhibition of viral replication among the 4 hmAbs, sustaining an inhibition rate of over 50% at 2 h and 6 h after infection. Overall, the findings indicate that the mechanism by which hmAbs neutralize SFTSV primarily affects virus binding and, to a lesser extent, virus internalization.

### hmAbs protect mice against a lethal SFTSV challenge

**Fig 5:**
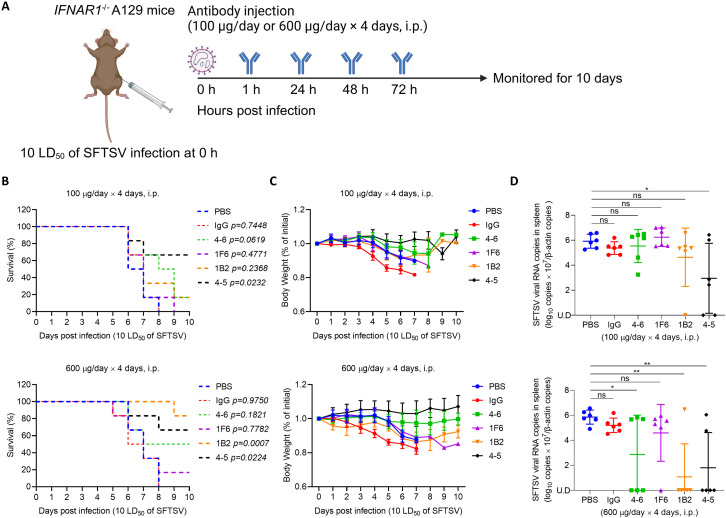
hmAbs protect mice against a lethal SFTSV challenge. (A) Six to eight weeks old *IFNAR1*^*-/-*^ A129 mice (n = 6 per group) were infected with 10 lethal doses (LD_50_) of SFTSV intraperitoneally. At 1, 24, 48, and 72 h post-infection, *IFNAR1*^*-/-*^ A129 mice were treated with 100 μg or 600 μg hmAb, human IgG control or PBS control intraperitoneally. The figure was created with BioRender.com. (B) The survival of *IFNAR1*^*-/-*^ A129 mice was monitored for 10 days after SFTSV infection. Kaplan–Meier survival curves were obtained using GraphPad Prism 8. (C) Body weight of mice was monitored daily for 10 days post-infection. Relative body weight values are presented as the mean with standard deviation of surviving mice in each group. (D) The SFTSV RNA copies in the mouse spleens were determined with RT-qPCR and were normalized by mouse *β*-actin. Two-tailed Student’s *t*-test was used to determine the level of statistical significance. The calculated *P*-values are shown above the groups that were compared. U.D., under the detection limit. Data are presented as mean ± standard deviation (SD). *, *P* < 0.05; **, *P* < 0.01; ***, *P* < 0.001; ****, *P* < 0.0001; ns, no significance.

Type I interferon (interferon α/β) receptor gene (IFNAR1)-deficient (*IFNAR1*^*-/-*^) A129 mice were considered as an animal model of SFTSV infection [34]. To further determine whether the hmAbs were protective to mice, *IFNAR1*^*-/-*^ A129 mice (n = 6 per group) were infected with 10 lethal doses (LD_50_) of SFTSV via intraperitoneal injection. At 1, 24, 48, and 72 h after SFTSV infection, each mouse was intraperitoneally injected with 100 or 600 μg hmAbs. PBS and human IgG were used as negative controls (Fig 5A). Mock-treated mice with PBS or human IgG all died within 8 days and lost 15 to 20% of body weight 5 to 7 days after SFTSV infection (Fig 5B). In contrast, the survival rate and body weight loss of mice treated with hmAbs depend on the hmAbs and their doses (Table 1). At a low dose group (100 μg/mouse/day), the hmAbs (4-6, 1B2), had partial protection on mice with a delayed mortality and survival rate of 16.7% (1/6). The hmAb 4-5 protected 66.7% (4/6) of mice against lethal SFTSV challenge. However, 1F6 presented no protection on the mice. In a high dose group (600 μg/mouse/day), treatment of 1F6 provided partially protection, with survival rates of 16.7% (1/6). The administration of hmAb (4-5 and 4-6) protected 66.7% (4/6) and 50% (3/6) of mice against lethal SFTSV challenge, respectively, without causing any noticeable weight loss in surviving mice. Noticeably, the hmAb 1B2 treated group, mice had a body weight reduction, but effectively protected 83.3% (5/6) of mice from lethal SFTSV infection (Fig 5B and 5C**).** The PBS group, the IgG group, and the low dose hmAb group exhibited notable pathological features, including atrophy or loss of white pulp in the spleens, and a large amount of SFTSV NP antigens were detected in the spleens. In contrast, the mice in the high dose mAb group did not show apparent pathological features in surviving mice, and only minor amounts of SFTSV NP antigens were detected in the spleens (S5 and S6 Figs). Furthermore, the viral RNA level in the spleens was significantly lower in living mice than in deceased mice, with some samples even falling below the detection limit. This effect was particularly pronounced in the high dose groups, providing compelling evidence for effective virus clearance following hmAbs treatment and demonstrating the strong neutralizing activity of these antibodies (Fig 5D). Collectively, these results suggest that the hmAbs 4-6, 1B2, and 4-5 could effectively protect mice against the lethal challenge of SFTSV.

**Table 1:** Survival rates of *IFNAR1*^*-/-*^ A129 mice treated with hmAbs against lethal SFTSV challenge.

Antibodies	Dose (µg/day)	Survival rate (%)
Low dose of hmAb		
PBS	–	0 (0/6)
Human IgG	100	0 (0/6)
4-6	100	16.7 (1/6)
1F6	100	0 (0/6)
1B2	100	16.7 (1/6)
4-5	100	66.7 (4/6)
High dose of hmAb		
PBS	–	0 (0/6)
Human IgG	600	0 (0/6)
4-6	600	50 (3/6)
1F6	600	16.7 (1/6)
1B2	600	83.3 (5/6)
4-5	600	66.7 (4/6)

### Pair combinations of hmAb fully protected mice from lethal SFTSV challenge

**Fig 6:**
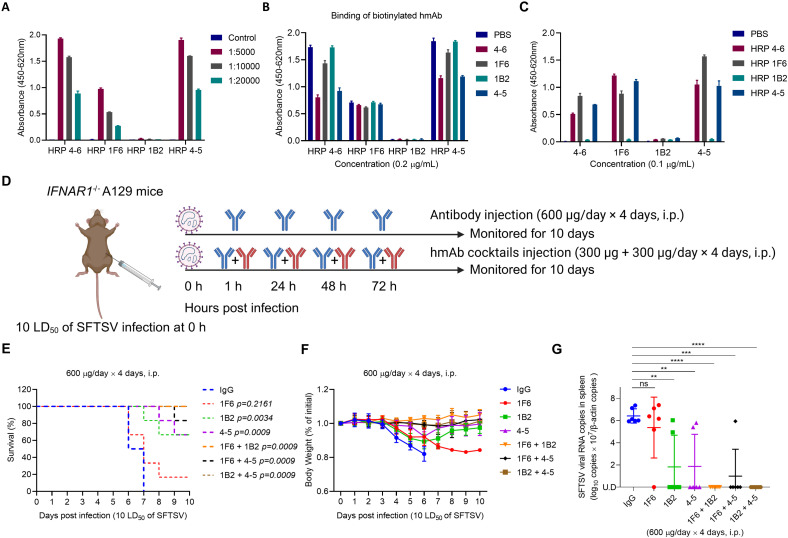
Pair combinations of hmAb fully protected mice from lethal SFTSV challenge. (A) Biotin-labeled (HRP-conjugated) hmAbs were detected with ELISA pre-coated with recombinant protein Gn. (B) Competitive ELISA was performed with 4 anti-Gn hmAbs. Biotinylated hmAbs were incubated with antigens (SFTSV Gn) in the presence of unlabeled competitor hmAbs, followed by detection. All experiments were performed in triplicate, and the data represented mean ± standard deviation. (C) Sandwich ELISA was performed using 4 unlabeled hmAbs as capture antibodies to coat 96-well EIA/RIA plates, followed by incubation with SFTSV Gn and biotin-labeled hmAbs. All experiments were performed in triplicate, and the data represented mean ± standard deviation. (D) Six to eight weeks old *IFNAR1*^*-/-*^ A129 mice (n = 6 per group) were infected with 10 LD_50_ of SFTSV intraperitoneally. At 1, 24, 48, and 72 h post-infection, *IFNAR1*^*-/-*^ A129 mice were injected intraperitoneally with individual antibodies or paired combinations of hmAbs 1F6, 1B2, and 4-5 at a total dose of 600 μg. Human IgG was used as control. The figure was created with BioRender.com. (E) Survival of *IFNAR1*^*-/-*^ A129 mice was monitored for 10 days. Kaplan–Meier survival curves were obtained using GraphPad Prism 8. (F) Body weight of mice was monitored daily for 10 days after SFTSV infection. Relative body weight values are presented as the mean with standard deviation of surviving mice in each group. (G) The SFTSV RNA copies in the mouse spleens were determined with RT-qPCR and were normalized by mouse *β*-actin. Two-tailed Student’s *t*-test was used to determine the level of statistical significance. The calculated *P*-values are shown above the groups that were compared. U.D., under the detection limit. Data are presented as mean ± standard deviation (SD). *, *P* < 0.05; **, *P* < 0.01; ***, *P* < 0.001; ****, *P* < 0.0001; ns, no significance.

Competitive ELISA and sandwich ELISA were used to determine whether the hmAbs recognize the same epitopes of Gn. The biotin-labeled hmAb was obtained through the covalent conjugation of hmAb to horseradish peroxidase (HRP). ELISA demonstrated that HRP-hmAbs 4-6, 1F6, and 4-5 exhibited comparable and high affinity for Gn in a dose-dependent manner, while HRP-1B2 did not bind to Gn (Fig 6A). Among the hmAbs 4-6, 1F6, and 4-5, competition ELISA showed that the binding of the biotinylated hmAb to Gn was attenuated to varying extents by its unlabeled counterpart. The binding of HRP hmAb 4-6 to Gn was reduced by hmAb 4-5, and vice versa, suggesting the hmAbs 4-6 and 4-5 recognized the same epitope on the SFTSV Gn protein (Fig 6B). Similarly, in sandwich ELISA, when 4-6 or 4-5 were used as capture antibodies, we found that the binding affinity of detection antibody 1F6 to Gn exceeded that of 4-6 and 4-5 when they served as detection antibodies (Fig 6C). Additionally, we also tried using 1F6 as the capture antibody. Interestingly, we found that the binding affinity of detection antibodies 4-6 and 4-5 to Gn was also higher than that of 1F6 (Fig 6C).

Three antibodies (hmAbs 1F6, 1B2, and 4-5) were mixed in pairs to test whether the monoclonal antibody combinations exert a cooperative protective effect in SFTSV-infected mice. *IFNAR1*^*-/-*^ A129 mice (n = 6 per group) were intraperitoneally infected with 10 LD_50_ of SFTSV and were then treated with each single hmAb (1F6, 1B2, or 4-5) or paired hmAb cocktails in the order of 1F6&1B2, 1F6&4-5, and 1B2&4-5 with a total antibody dose of 600 μg each mouse at 24 h intervals for 4 days (Fig 6D). The control group was treated with human IgG, all mice died within 7 days and lost approximately 15–20% of body weight 4–6 days after SFTSV infection. hmAb cocktails treatment significantly improved the antibody protective effect on mice compared to single antibody treatment groups. In the single antibody treatment groups, hmAb 1F6 displayed 16.7% (1/6) protection, while 66.7% (4/6) of mice treated with hmAb 1B2 or hmAb 4-5 survived. In the paired hmAb cocktails, mice survival rates ranged from 83.3% to 100% across different antibody combinations. The groups treated with the hmAb cocktails of hmAb 1F6 and 1B2 or 1B2 and 4-5 were able to protect 100% (6/6) of mice from the lethal SFTSV challenge. Treatment of the cocktail of hmAb 1F6 and 4-5 provided 83.3% (5/6) protection (Table 2 and Fig 6E). In addition, all mice treated with hmAb cocktails did not have body-weight loss (Fig 6F). Moreover, the viral RNA levels in the spleens of surviving mice were effectively cleared compared with dead mice in control group (Fig 6G). Taken together, these results indicate the clear cooperative effects of the three pair combinations of hmAbs (1F6, 1B2, and 4-5) in the protection of mice against the SFTSV challenge.

**Table 2:** Survival rates of *IFNAR1*^*-/-*^ A129 mice treated with hmAb cocktails against lethal SFTSV challenge.

Antibodies	Dose (µg/day)	Survival rate (%)
Human IgG	600	0 (0/6)
1F6	600	16.7 (1/6)
1B2	600	66.7 (4/6)
4-5	600	66.7 (4/6)
1F6&1B2	300&300	100 (6/6)
1F6&4-5	300&300	83.3 (5/6)
1B2&4-5	300&300	100 (6/6)

## Discussion

Currently, we are facing numerous emerging infectious disease outbreaks worldwide, such as COVID-19 outbreaks [35], which have caused severe global panic and lack of specific treatments. Similarly, the SFTSV outbreak also poses a serious threat to public health, especially in Asian countries. Antibodies have emerged as a promising approach to combat various viral infections, particularly for the rapidly responding to emerging viruses, such as HIV-1, Ebola, SARS-COV-2, and ZIKV [36-40]. Antibody therapies for treating SARS-CoV-2 have been approved for clinical use [30,41,42]. Current studies indicated that antibody combined application has made remarkable achievements in the antiviral field, this kind of therapy has been used in a variety of viruses, such as the Ebola virus, SARS-CoV-2, and respiratory syncytial virus [29-31]. A previous study showed that combination therapy with two monoclonal antibodies against *Bunyavirales* Rift Valley fever virus (RVFV) provided complete protection by cooperative effects in BALB/c mice [43]. A previous study showed that cocktails of neutralizing antibodies recognizing different antigen epitopes cooperatively neutralize SARS-CoV-2 and optimize antibody therapy efficacy [44]. Developing neutralizing antibodies and antibody cocktails is crucial for combating the life-threatening SFTSV infection and improving clinical treatment outcomes. However, no antibody cocktails have been tested for treating SFTSV infection.

In this study, we tested hmAbs, 4-6, 1F6, 1B2, and 4-5 derived from phage display libraries of convalescent SFTS patients for neutralization of SFTSV in cell culture and protection of mice against lethal challenge of SFTSV [32]. We found that 4-5, 1F6, and 1B2 recognized distinct Gn epitopes and tested the protectivity of the hmAbs individually and at pair combinations in *IFNAR1*^*-/-*^ A129 mice. We found that all hmAbs had the potential to protect mice against lethal SFTSV challenge with the survival rate of mice from 16.7% to 83.3% compared to 100% death in mice without hmAb. Pair combinations of hmAbs 1F6 and 1B2 or 1B2 and 4-5 significantly improved the survival rate of mice and both pair combinations can fully protect *IFNAR1*^*-/-*^ A129 mice against the lethal SFTSV challenge. Furthermore, we also demonstrated that, compared with single antibodies, hmAb cocktails not only improved the survival rate of mice but also eliminated body weight loss and promoted virus clearance in SFTSV-infected mice, further proving the potency of these hmAb cocktails in the treatment of SFTSV infection. Our study indicated that these two pairs of hmAbs have a promising future for the treatment of SFTSV infection in humans.

Our study indicated that treatment with hmAbs 4-6, 1B2, and 4-5 provided effective protection against SFTSV lethal challenge in terms of survival and changes in body weight in mice compared to 1F6. The effect of hmAb 1F6 *in vitro* and *in vivo* differed significantly, which may be influenced by multiple factors. *In vitro*, hmAb 1F6 directly engages with viral particles. However, *in vivo*, diverse immune regulatory factors, stability, and half-life may influence its effectiveness, leading to rapid degradation or elimination and insufficient effective concentration [45]. In addition, the CDR regions of hmAb 1F6 may exhibit stronger immunogenicity and elicit a more robust immune response in mice, potentially impacting its therapeutic effect [46].

**Fig 7:**
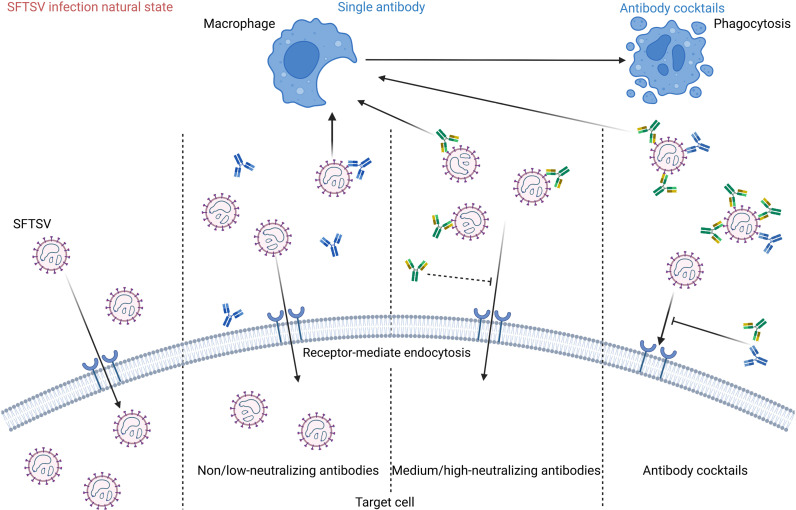
Schematic model illustrating the process of antibody neutralizing SFTSV in vivo. Neutralizing antibodies can bind to virus particles, prevent their attachment to host cell surface receptors, and thereby block the virus from entering host cells. Following viral neutralization, they stimulate macrophages to engulf and degrade the virus. Non/low-neutralizing antibodies binding to Gn can induce the conformational change, making the neutralization epitope of medium/high-neutralizing antibodies being more accessible, thus enhancing the neutralizing activity. In addition, the antibody cocktails achieved more efficient clearance by targeting different neutralizing epitopes on the viral Gn protein. The model was created with BioRender.com.

mAb is widely recognized as an effective means for neutralizing pathogens and treating viral diseases. However, as an RNA virus, SFTSV may evade neutralization through accumulation mutations. A phylogenetic analysis of SFTSV isolates revealed substantial genetic diversity and cumulative mutations, potentially affecting single mAb therapy efficacy [47,48]. Given these challenges, developing therapeutic mAb cocktails have emerged as a promising strategy to address virus-associated antibody resistance and escape mutations. Using multiple mAbs targeting different neutralization sites in combination can produce complementary or cooperative effects, ensuring strong protection against viral variants. So far, no research on hmAb cocktails against SFTSV has been reported. Our study revealed that hmAbs 1F6, 1B2, and 4-5 target distinct epitopes, and exhibited a cooperative protective effect in mice against SFTSV lethal challenge when they were paired together. The mechanisms of the enhanced protectivity of the pair hmAbs in this study are not clear. Previous studies reported that the underlying mechanism for the observed “cooperative neutralization” originates from the specific binding of a single antibody to envelope glycoprotein, which initiates conformational changes of antigens, enhancing the accessibility of epitopes for other mAbs, thus allowing for a more comprehensive and effective binding [43,49]. Similar cooperative protective effects of neutralizing mAbs improved by other non-neutralizing monoclonal antibodies have been described for mAbs against Ebola virus, HIV and RVFV [43,49,50]. The individual use of medium-neutralizing hmAb 1B2 and 4-5 can protect over 50% of mice from fatal SFTSV infection. After the combination with low-neutralizing hmAb 1F6, 83.3% of mice with the combination of hmAb 1F6 and 4-5 group survived, while the treatment of the hmAb 1F6 and 1B2 group provided complete protection (Fig 7). Similarly, pair combinations of hmAb 1B2 and 4-5 also provided complete protection of mice against the lethal dose of SFTSV challenge.

Previous studies reported several anti-Gn mAbs had neutralizing activities against SFTSV infection in cells and animals. Camel-derived SNB02 and mouse-derived MAb40C10 displayed a potent therapeutic effect in NCG-HuPBL mice or C57BL/6 mice, respectively [26,27]. Several hmAbs were reported to have neutralization potency *in vitro* and/or therapeutically effective in *IFNAR1*^*-/-*^ A129 mice. However, M1-B8, M1-D1, M1-E1, and M1-E5 showed a relatively low therapeutic effect with no more than 20% protection [51]. Ab10 and SF5 could provide complete protection in lethal SFTSV-challenged *IFNAR1*^*-/-*^ A129 mice [25,28].

In this study, we discovered that all hmAbs tested effectively bound the virus well according to the Western blot. The absence of reactivity between hmAb 1B2 and Gn protein in ELISA tests suggests structural alterations in the full-length monoclonal antibody expressed in eukaryotic cells compared to scFv. However, the exact underlying mechanisms behind this phenomenon remain unclear.

We have demonstrated that the hmAbs can neutralize distinct genotypes of SFTSV, revealed that the hmAbs exhibited a pronounced neutralization effect across diverse viral genotypes. The limitation of our study is that we did not test whether viruses can escape the hmAb neutralization in cell culture and in animals.

In summary, we found that pair combinations of hmAbs 1B2 and 1F6, and 1B2 and 4-5 could fully protect mice against lethal SFTS challenge indicating these pair combinations of human monoclonal antibodies may be used to treat SFTSV-infected humans.

## Materials and methods

### Ethics statement

All mice used in our study were housed with free access to food and water under a 12:12 light-dark cycle. The study was reviewed and approved by the ethics committee of Wuhan University (WHU2018010). *IFNAR1*^*-/-*^ A129 mice were bred and maintained in a specific pathogen-free animal facility. The body weight of the mice (six to eight weeks old) ranged from 17g to 23g with an average body weight of 20g. The selected mice were acclimated for at least 1 week and the mice were not subjected to inappropriate handling to prevent any form of undue stress or discomfort before any procedures were carried out.

The procedures for animal experiments followed previous reports [25,27,28]. For *in vivo* infection experiments, a double-blind randomized approach was employed. Six to eight weeks old *IFNAR*^*-/-*^ A129 mice were randomly divided into hmAb-treatment and control groups, with 3 males and 3 females in each group. The mice were infected with 10 LD_50_ of SFTSV via intraperitoneal injection. At 1, 24, 48, and 72 h after SFTSV infection, each mouse in the hmAb-treatment group was intraperitoneally injected with 100 or 600 µg single hmAb, or a hmAb cocktail (600 µg), while the control group was intraperitoneally injected with an equivalent volume of PBS or equal weight of IgG. Mice were monitored daily for weight loss and survival. On day 10, surviving mice were anesthetized using a mixture of 100 µL of ketamine (150 mg/kg) and xylazine (15 mg/kg). Following anesthesia, the mice were euthanized humanely using inhaled carbon dioxide. Immediately after death or euthanasia, the spleens were excised from each animal, and RNA was extracted from splenocytes for subsequent viral load analysis.

### Cells and viruses

Vero and HEK293T cells were cultured in Dulbecco’s modified Eagle’s medium (DMEM; Gibco, Beijing, China) with 10% fetal bovine serum (FBS; Gibco, Auckland, New Zealand) and 1% penicillin-streptomycin. All cells were cultured at 37°C with 5% CO_2_. SFTSV strain JS2011-013-1, representing clades I [33], was obtained from the Jiangsu Provincial Center for Disease Prevention and Control. SFTSV strains HNXY2017-66, HBMC16_human_2015, and HNXY2017-50, representing clades II, III, and IV, respectively [33], were kindly provided by Dr. Lei-Ke Zhang (Wuhan Institute of Virology, Chinese Academy of Sciences, China). All the viruses were propagated in Vero cells. Supernatants containing SFTSV viral particles were harvested and stored at -80°C. The fifty-percent tissue culture infectious doses (TCID_50_) were titrated on Vero cells based on the Reed-Muench method.

### Screening of human SFTSV monoclonal antibodies

Human peripheral blood lymphocytes were collected from 5 convalescent SFTS patients in Jiangsu Province and all participants provided written informed consent. Total RNA was isolated and cDNA was synthesized using a first-strand cDNA synthesis kit (Invitrogen, CA, USA) with oligo (dT) priming. Based on the cDNA, a human phage-display library with single-chain variable fragments format (scFv) was constructed, and four rounds of biopanning were performed to select phages from the library, as previously described [32,52,53]. After the 4th round of panning, the eluted phage colonies were tested for binding to SFTSV glycoprotein Gn in a phage enzyme-linked immunosorbent assay (ELISA). The nucleotide sequences of variable region genes of heavy (VH) and light (VL) chains from positive clones were sequenced.

### Eukaryotic expression of SFTSV glycoprotein Gn/Gc and human monoclonal antibodies

The recombinant eukaryotic expression plasmids pCAGGS-Gn-strepII and pCAGGS-Gc-strepII containing Gn (GenBank accession No. JF906057.1, residues 20-452) and Gc (GenBank accession No. JF906057.1, residues 563-1036) fragments were constructed, respectively. The recombinant eukaryotic expression plasmids Gn and Gc were transfected into HEK293T cells for expression. Cell supernatants were collected after transfection. The engineering and production of the human immunoglobulin G4 (IgG4) was performed as described previously. The VH and VL regions of the scFv-positive clone, were PCR amplified using specific primers, fused with the constant regions (IgG4) to form a complete heavy and light chain structure by overlap PCR, and then cloned into the pCAGGS eukaryotic expression vector. The recombinant expression plasmids (VH: VL=30 µg: 20 µg) were transfected into HEK293T cells using polyethyleneimine (150 µg). The supernatant containing recombinant protein was collected at 72 h after transfection.

### Purification of SFTSV glycoprotein Gn/Gc and human monoclonal antibodies

Cell supernatants of HEK293T cells transfected with recombinant plasmids pCAGGS-Gn-strepII and pCAGGS-Gc-strepII were centrifuged (at 8000×*g* for 90 min), mixed with 20 mM Tris-150 mM NaCl in a 1:1 ratio, and filtered through a 0.22 µM filter membrane. The solution was loaded onto a StrepTrap HP column (GE Healthcare, Uppsala, Sweden) and eluted with buffer A (20 mM Tris, 150 mM NaCl, pH = 8.0) and buffer B (20 mM Tris, 150 mM NaCl, and 5 mM Desthiobiotin, pH = 8.0). The eluate was purified using HiLoad Superdex 200 pg column (Cytiva, Marlborough, USA).Supernatants of HEK293T cells transfected with light and heavy chain recombinant plasmids were centrifuged (8000×*g*, 90 min) and mixed with 20 mM Na_3_PO4 at 1:1. The solution was filtered through a 0.22 µM filter membrane and loaded onto a HiTrap Protein A column (GE Healthcare). The hmAbs were eluted with 0.1 M glycine (pH = 3.0), and the eluate was purified using HiLoad Superdex 200 pg column (Cytiva). The purified protein was kept at -80°C until use.

### Elisa

First, 100 ng bovine serum albumin (BSA), recombinant SFTSV Gn and Gc proteins were coated on the 96-well EIA/RIA plates (Corning, New York, USA) overnight, and blocked with 1% BSA for 1 h. After washing with PBST (PBS with 0.05% Tween-20) 5 times, 100 μL of hmAbs (0.1 μg/mL) were added to each well and incubated for 1 h. The plates were washed with PBST 5 times and incubated with horseradish peroxidase (HRP)-conjugated anti-human antibody (Proteintech, Wuhan, China) for 1 h. The plates were washed with PBST 5 times, and each well was added 50 μL of 3,3′,5,5′-tetramethylbenzidine (TMB) substrate (Solarbio, Beijing, China). The reaction was stopped with 50 μL of 2 M sulfuric acid. The absorbance was measured at 450 nm and 620 nm using a microplate reader (Tecan).

### Median tissue culture infectious dose (TCID_50_)

The SFTSV virus solution was serially diluted ten-fold and each dilution was add to 8–16 wells of Vero cells in a 96-well plate. After 24 h incubation, the monolayers were fixed with 4% paraformaldehyde for 30 min and permeabilized with 0.2% Triton-100 for 15 min. After blocking with 1% BSA, the cells were incubated with primary antibodies to SFTSV NP at 4°C overnight; followed by incubating with FITC-labeled Goat Anti-Human IgG (Proteintech) for 30 min. Nuclei were stain with DAPI (Life-iLab, Shanghai, China) for 10 min. All cells were washed with PBS 3times after each step. The fluorescence was observed under a fluorescence microscope. The virus titer was calculated with Reed Muench’s method.

### Neutralization test

The virus solution (100 TCID_50_), was mixed with a hmAb dilution at 1:1 ratio and incubated at 37°C for 1 h. Subsequently, the mixture of virus and antibodies were seeded on the cells and incubated at 37°C for 2 h before being replaced by maintenance medium for another 24–72 h. The cells were harvested for subsequent experiments.

### Western blot analysis and antibodies

Cells were lysed with RIPA Lysis Buffer (Beyotime, Shanghai, China) containing protease inhibitor cocktail. Protein samples were briefly ultrasonicated for 30 s, heated for 10 min at 95°C, separated with SDS-PAGE (80 V for 30 min, 120 V for 1 h), and transferred to the PVDF membrane (Cytiva) (200 mA for 2 h). Membranes were blocked with 5% non-fat milk in Tris-buffered saline and Tween 20 (TBST) for 1 h. Membranes were incubated with primary antibodies overnight at 4°C and HRP-labeled secondary antibodies for 2 h. Protein bands were imaged in the Amersham Imager 600 system. Anti-SFTSV NP monoclonal antibody was maintained in our laboratory. Mouse anti-*β*-actin monoclonal antibody (K200058M) was purchased from Solarbio. HRP-conjugated goat anti-mouse (SA00001-1) and goat anti-human (SA00001-17) were purchased from Proteintech.

### RNA isolation and real-time quantitative PCR

Total RNA was isolated with TRIzol Reagent (Invitrogen, Carlsbad, CA) and transcribed into cDNA with the NovoScript^®^ Plus All-in-one 1st Strand cDNA Synthesis SuperMix (gDNA Purge) (Novoprotein, Shanghai, China; E047). RT-qPCR assays were performed using a ChamQ Blue Universal SYBR qPCR Master Mix (Vazyme, Nanjing, China). Data were normalized to the *gapdh* mRNA level and relative mRNA concentrations were calculated with the 2^−ΔΔCt^ method. The primers are listed in Table 3.

**Table 3:** Primers used for RT-qPCR.

Genes	Forward	Reverse
SFTSV L	AGTCTAGGTCATCTGATCCGTTTAG	TGTAAGTTCGCCCTTTGTCCAT
SFTSV M	AAGAAGTGGCTGTTCATCATTATTG	GCCTTAAGGACATTGGTGAGTA
SFTSV S	TGTCAGAGTGGTCCAGGATT	ACCTGTCTCCTTCAGCTTCT
Human GAPDH	GGAGCGAGATCCCTCCAAAAT	GGCTGTTGTCATACTTCTCATGG
Mouse β-actin	GTGCTATGTTGCTCTAGACTTCG	ATGCCACAGGATTCCATACC

### SFTSV binding assay

Viruses (1 MOI, 100 µL) were premixed with 100 µg of hmAb (100 µL) or an equal volume of PBS for 1 h, then was added to ice cold Vero cells and incubated at 4°C for 1 h. After washed with ice cold PBS 3 times, total RNA was extracted, and the RNA level of bound viral particles was quantified with RT-qPCR.

### SFTSV internalization assay

Ice-cold Vero cells were added SFTSV (1MOI, 100 µL) on ice and incubated at 4°C for 1 h. The cells were washed with ice-cold PBS to remove extra virus, and 100 µg hmAb in 100 µL or an equal volume of PBS was added to the ice-cold cells, and cells were incubated at 37°C for 2 h. Cells were then washed with PBS and treated with trypsin to remove unbound and bound viruses to the membrane. The RNA load of internalized SFTSV was determined with RT-qPCR.

### Dynamics of hmAb neutralizion

To determine the effective time points of hmAb neutralization, Vero cells were infected with SFTSV (1 MOI) and incubated at 37°C with 5% CO_2_. hmAb (100 µg) or NH4Cl (30 mM) as control was added to the cells 12 h before virus infection (−12 h), during infection (0 h), or after infection (2, 6, and 12 h), respectively. Two hours after virus infection, the cells were washed twice with PBS and incubated with fresh medium containing 100 µg hmAb at the indicated time points. Total protein of the cells was analyzed with Western blot and total RNA was extracted from the cells for measuring viral RNA load with RT-qPCR 24 h after virus infection.

### Competitive ELISA

hmAbs were conjugated with HRP coupling kit (Abcam, Cambridge, UK). SFTSV Gn (1 µg/mL) were used as antigens to coat 96-well EIA/RIA plates (Corning) overnight. Following coating, the plates were washed 5 times with PBST, and blocked with 1% BSA for 1 h. SFTSV Gn antigens were incubated with unlabeled hmAbs or PBS at 0.2 µg/mL for 30 min, followed by incubation with biotin-labeled hmAbs at 0.2 µg/mL for 30 min. After washing, colorization was performed with 50 µL of TMB (Solarbio), and then terminated with 50 µL of 2M sulfuric acid. Dual-wavelength determinations were performed with 450 nm and 620 nm using a microplate reader (Tecan).

### Sandwich ELISA

Unlabeled hmAbs (1 µg/mL) were used as capture antibodies to coat 96-well EIA/RIA plates (Corning) overnight. The plates were washed 5 times with PBST, and blocked with 1% BSA for 1 h, and washed 5 times with PBST. The plates were incubated with SFTSV Gn (1 µg/mL) for 1 h and then washed 5 times with PBST. Biotin-labeled hmAbs (0.1 µg/mL) were added to the plates and incubated for 1 h. After washing, each well was incubated with 50 µL of TMB (Solarbio) for 10 min. The color reaction was terminated with 50 µL of 2M sulfuric acid. The absorbance was read with a microplate reader (Tecan) at 450 nm and 620 nm to measure.

### Statistical analysis

Most experiments were performed 3 times. All data was analyzed using GraphPad Prism 8.0. Student’s *t*-test was used for statistical analysis. Survival rates were calculated using the Kaplan–Meier method, where *P* < 0.05 was considered statistically significant (*, *P* < 0.05; **, *P* < 0.01; ***, *P* < 0.001; ****, *P* < 0.0001; ns, no significance).

## Supporting information

S1 Fig Purification and SDS-PAGE analysis of purified glycoprotein Gn and Gc. (A) The glycoprotein Gn (residues 20-452) was expressed in HEK293T cells using the eukaryotic expression vector pCAGGS. The recombinant plasmid pCAGGS-Gn-strepII was transfected into HEK293T, and the supernatant was harvested and purified with StrepTrap HP column and HiLoad Superdex 200 pg column, followed with SDS-PAGE and Coomassie brilliant blue staining of each tube of eluate in sequence based on the peak locations. (B) The glycoprotein Gc (residues 563-1035) was expressed in HEK293T cells using the eukaryotic expression vector pCAGGS. The purification procedure is the same as the glycoprotein Gn.
(TIF)

S2 Fig Production and identification of hmAbs to SFTSV Gn. (A-C) The recombination of the light and heavy chains of human monoclonal antibodies eukaryotic expression plasmid was transfected into HEK293T, the supernatant was harvested and antibodies were purified from the culture supernatant using HiTrap Protein A column and HiLoad Superdex 200 pg column, followed with SDS-PAGE and Coomassie brilliant blue staining of each tube of eluate in sequence under reducing (right) and nonreducing conditions (left) based on the peak locations. The heavy and light chains of hmAbs are approximately 55 kDa and 25 kDa, respectively.
(TIF)

S3 Fig Neutralizing effect of hmAbs against three different SFTSV strains. (A-C) Each hmAb (25 µg/mL) was premixed with 100 TCID_50_ of three different SFTSV strains (HNXY2017-66, HBMC16_human_2015, and HNXY2017-50) at 37°C for 1 h, and the mixture was incubated with cells for 2 h, and then the supernatant was replaced with 2% maintenance medium and cultured at 37°C for 24–48 h. RT-qPCR was used to determine the viral RNA level of SFTSV.
(TIF)

S4 Fig Dynamics of NH_4_Cl neutralizing. NH_4_Cl (30mM) was added to cells before SFTSV infection (−12 h), during SFTSV infection (0 h), or after SFTSV infection (2, 6, and 12h) at different time points. The cells were harvested 24 h after SFTSV infection and the protein level of SFTSV NP were analyzed with Western blot. Total cell RNA was prepared 24 h after SFTSV infection and the level of viral RNA was analyzed with RT-qPCR.
(TIF)

S5 Fig Immunohistochemical analysis of spleens of mice challenged with SFTSV and treated with hmAbs. Immunohistochemical staining of spleens of mice (*IFNAR1*^*-/-*^ A129) which were challenged with lethal doses of SFTSV and post-treated with hmAbs. SFTSV NP antigen (brown) and cell nucleus (blue) in tissues were detected. Bar:100 μm.
(TIF)

S6 Fig Histopathologic analysis of spleens of mice challenged with SFTSV and treated with hmAbs. After challenging with lethal doses of SFTSV and treatment with hmAbs, the spleens of *IFNAR1*^*-/-*^ A129 mice were stained with hematoxylin-eosin to observe histopathologic changes. Bar:100 μm.
(TIF)
