# Supplementary figures and images for: Pair combinations of human monoclonal antibodies fully protected mice against bunyavirus SFTSV lethal challenge
Source: PLoS Pathog. 2025 Jan 31;21(1):e1012889. doi: 10.1371/journal.ppat.1012889 (PMC11785279; doi:10.1371/journal.ppat.1012889)

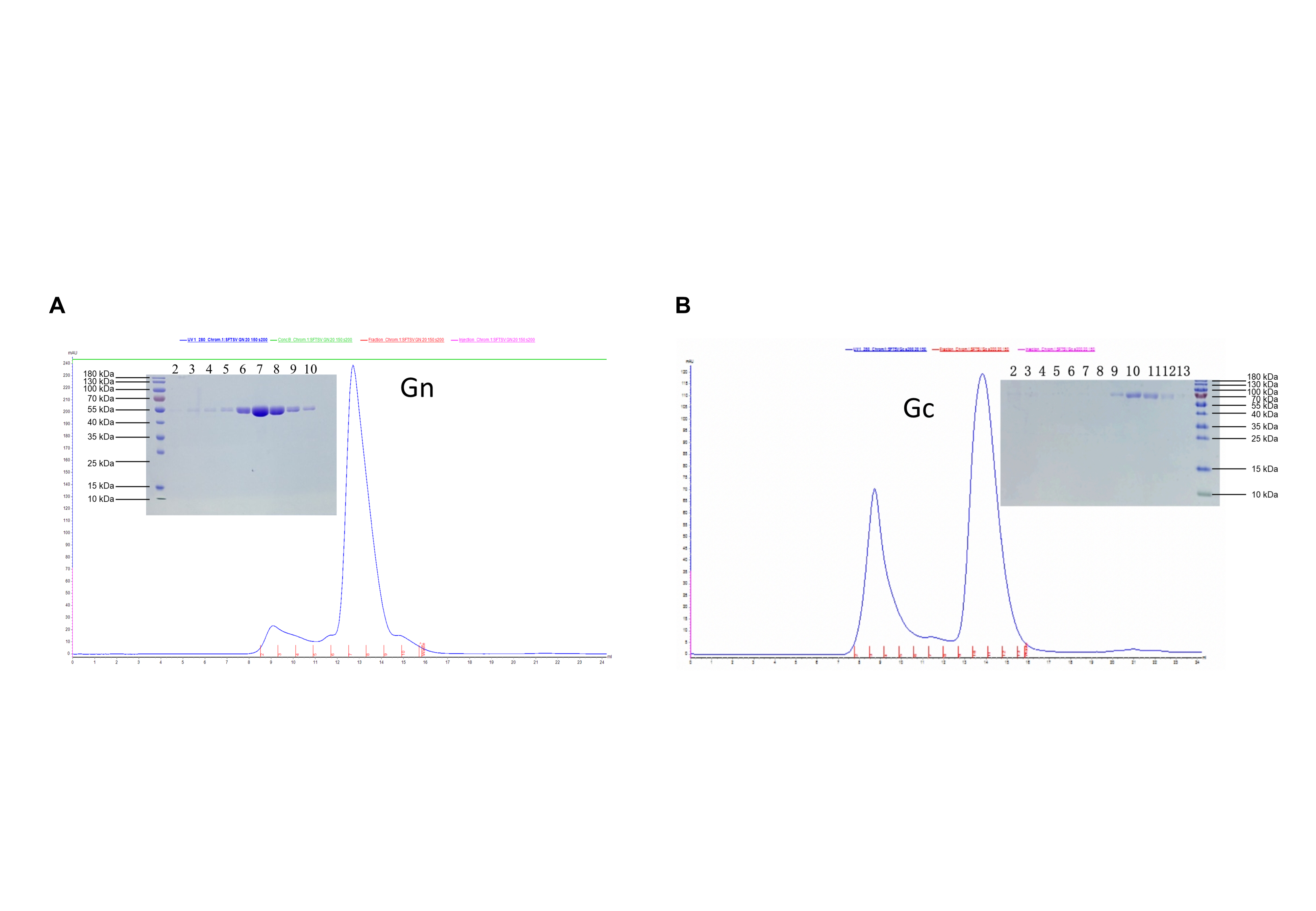

Supplement: S1 Fig — (A) The glycoprotein Gn (residues 20-452) was expressed in HEK293T cells using the eukaryotic expression vector pCAGGS. The recombinant plasmid pCAGGS-Gn-strepII was transfected into HEK293T, and the supernatant was harvested and purified with StrepTrap HP column and HiLoad Superdex 200 pg column, followed with SDS-PAGE and Coomassie brilliant blue staining of each tube of eluate in sequence based on the peak locations. (B) The glycoprotein Gc (residues 563-1035) was expressed in HEK293T cells using the eukaryotic expression vector pCAGGS. The purification procedure is the same as the glycoprotein Gn. (TIF) [file ppat.1012889.s001.tif]

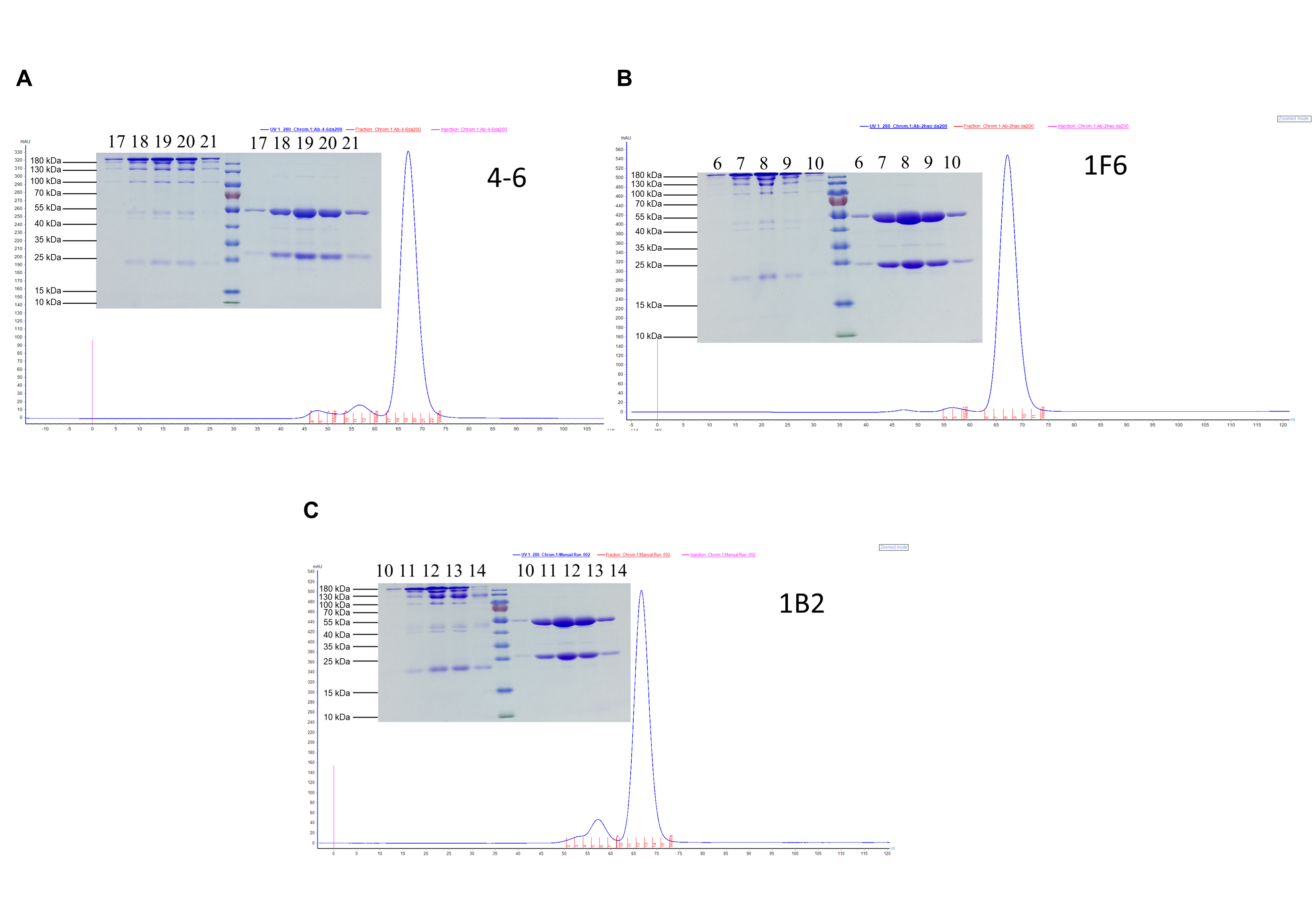

Supplement: S2 Fig — (A-C) The recombination of the light and heavy chains of human monoclonal antibodies eukaryotic expression plasmid was transfected into HEK293T, the supernatant was harvested and antibodies were purified from the culture supernatant using HiTrap Protein A column and HiLoad Superdex 200 pg column, followed with SDS-PAGE and Coomassie brilliant blue staining of each tube of eluate in sequence under reducing (right) and nonreducing conditions (left) based on the peak locations. The heavy and light chains of hmAbs are approximately 55 kDa and 25 kDa, respectively. (TIF) [file ppat.1012889.s002.tif]

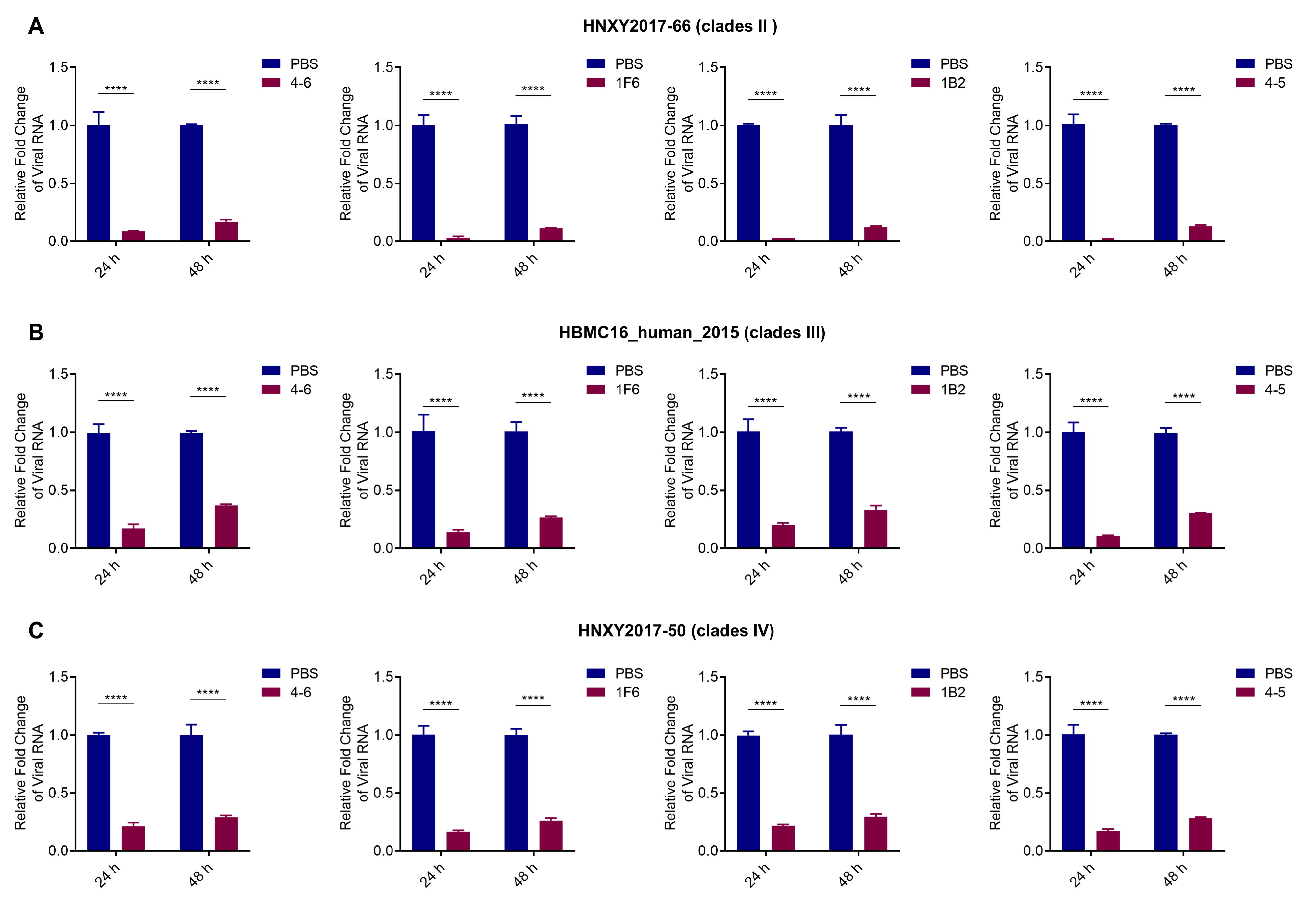

Supplement: S3 Fig — (A-C) Each hmAb (25 µg/mL) was premixed with 100 TCID50 of three different SFTSV strains (HNXY2017-66, HBMC16_human_2015, and HNXY2017-50) at 37°C for 1 h, and the mixture was incubated with cells for 2 h, and then the supernatant was replaced with 2% maintenance medium and cultured at 37°C for 24–48 h. RT-qPCR was used to determine the viral RNA level of SFTSV. (TIF) [file ppat.1012889.s003.tif]

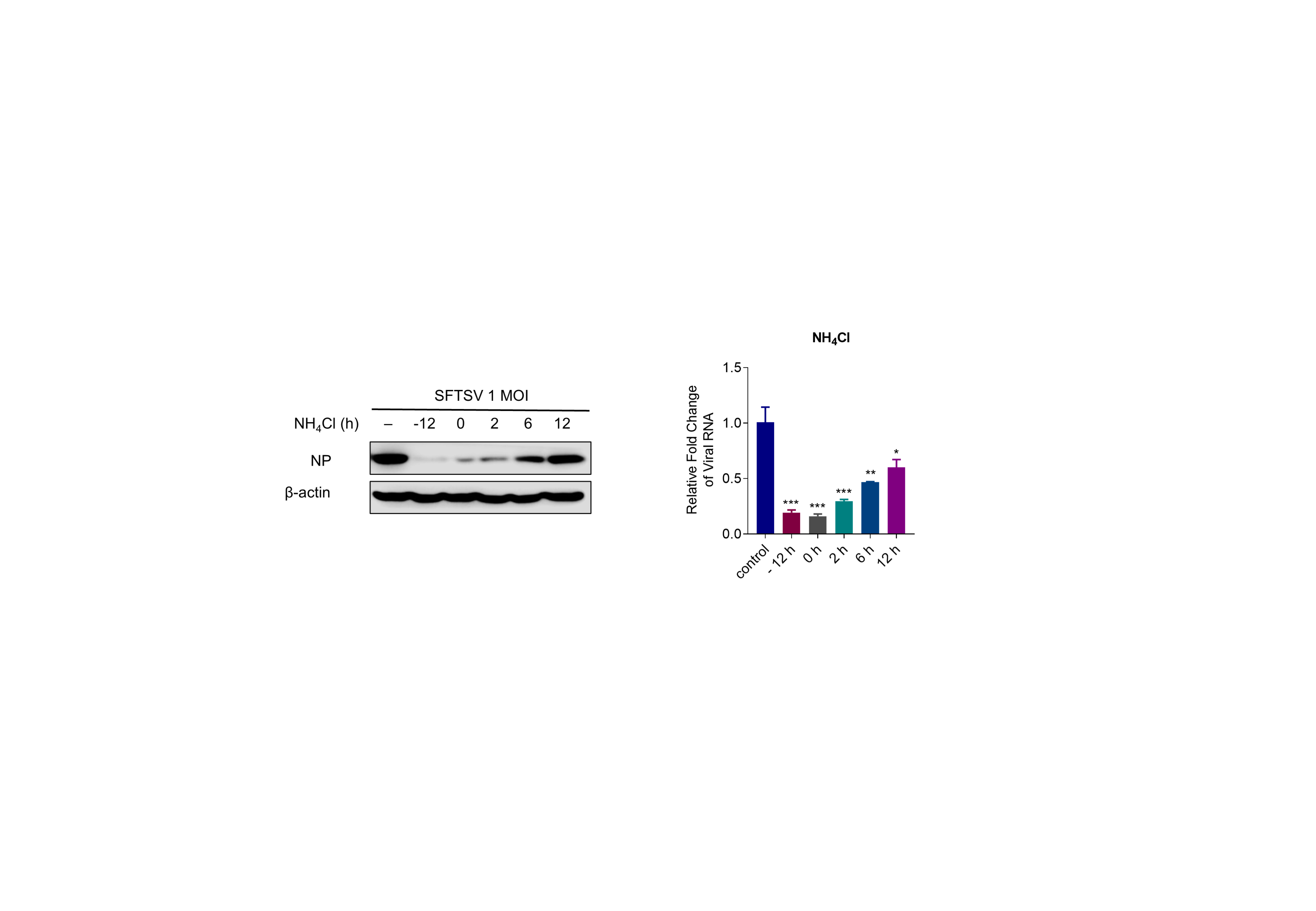

Supplement: S4 Fig — NH4Cl (30mM) was added to cells before SFTSV infection (−12 h), during SFTSV infection (0 h), or after SFTSV infection (2, 6, and 12h) at different time points. The cells were harvested 24 h after SFTSV infection and the protein level of SFTSV NP were analyzed with Western blot. Total cell RNA was prepared 24 h after SFTSV infection and the level of viral RNA was analyzed with RT-qPCR. (TIF) [file ppat.1012889.s004.tif]

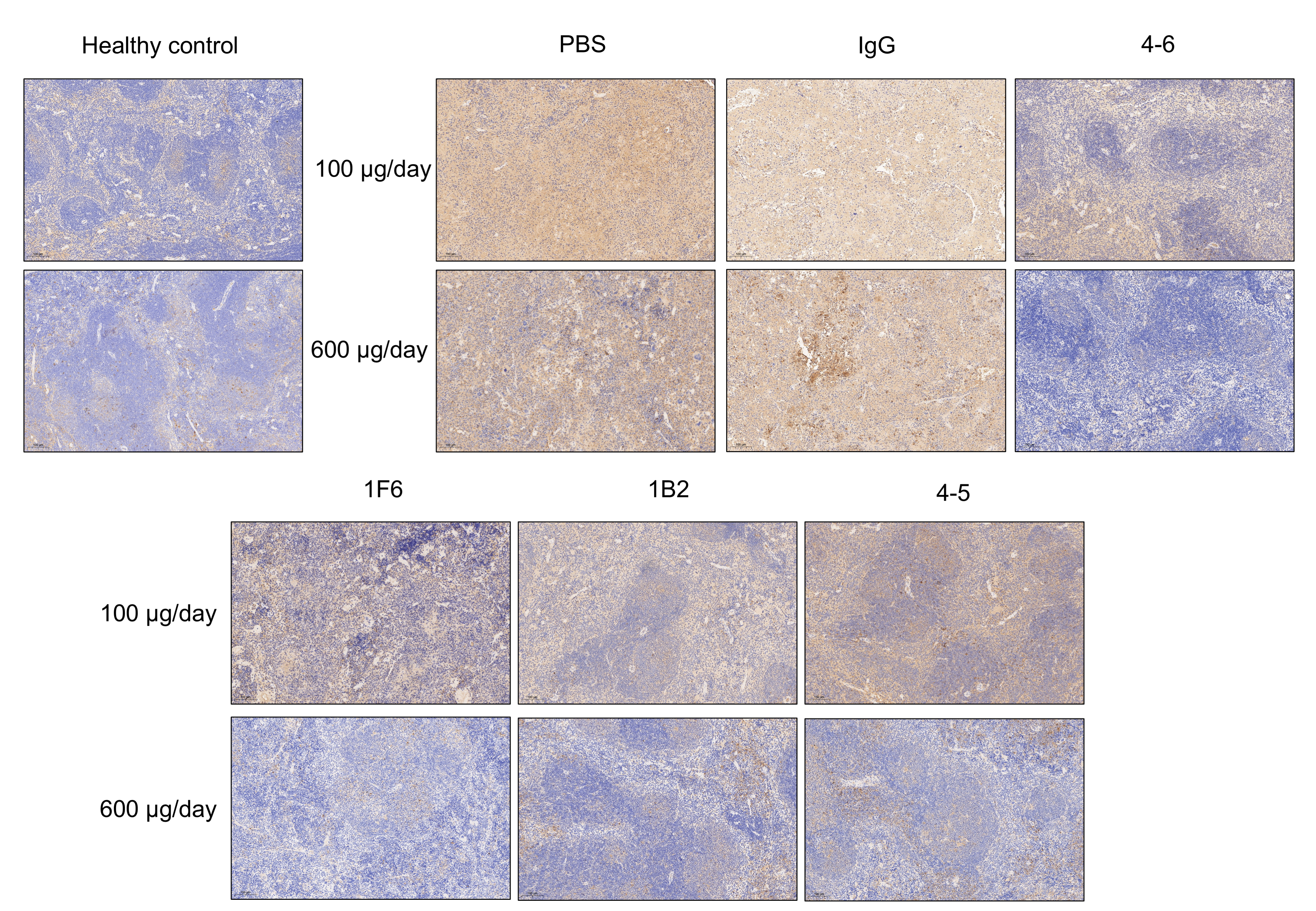

Supplement: S5 Fig — Immunohistochemical staining of spleens of mice (IFNAR1-/- A129) which were challenged with lethal doses of SFTSV and post-treated with hmAbs. SFTSV NP antigen (brown) and cell nucleus (blue) in tissues were detected. Bar:100 μm. (TIF) [file ppat.1012889.s005.tif]

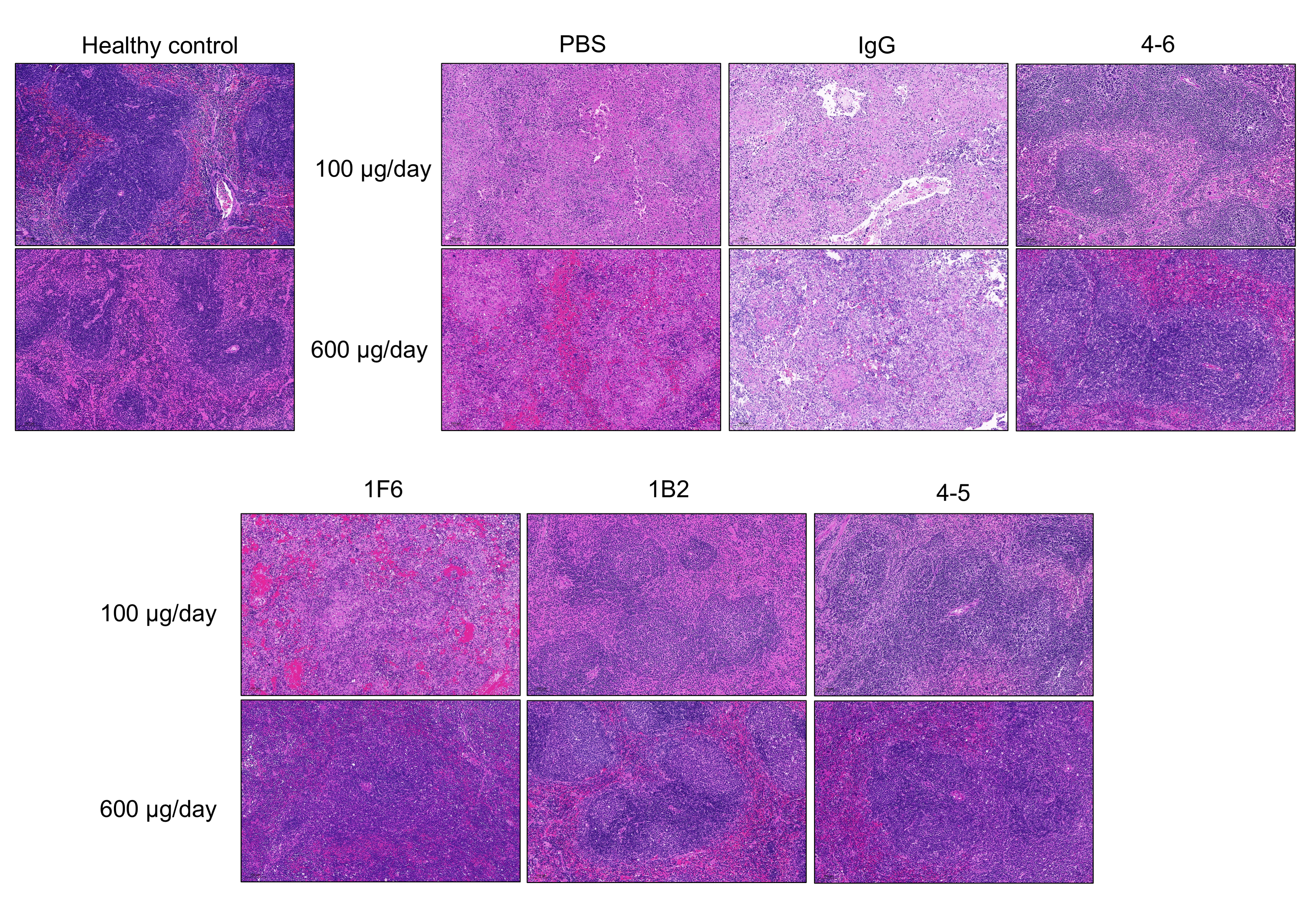

Supplement: S6 Fig — After challenging with lethal doses of SFTSV and treatment with hmAbs, the spleens of IFNAR1-/- A129 mice were stained with hematoxylin-eosin to observe histopathologic changes. Bar:100 μm. (TIF) [file ppat.1012889.s006.tif]
